# Supplementary material for: Relationship between Amino Acid Metabolism and Bovine In Vitro Follicle Activation and Growth
Source: Animals (Basel). 2023 Mar 23;13(7):1141. doi: 10.3390/ani13071141 (PMC10093075; doi:10.3390/ani13071141)
Supplement: Supplementary file 1 [file animals-13-01141-s001.zip › Sakaguchi et al. Animals Supplemental figure revised.pptx]

## Slide 1
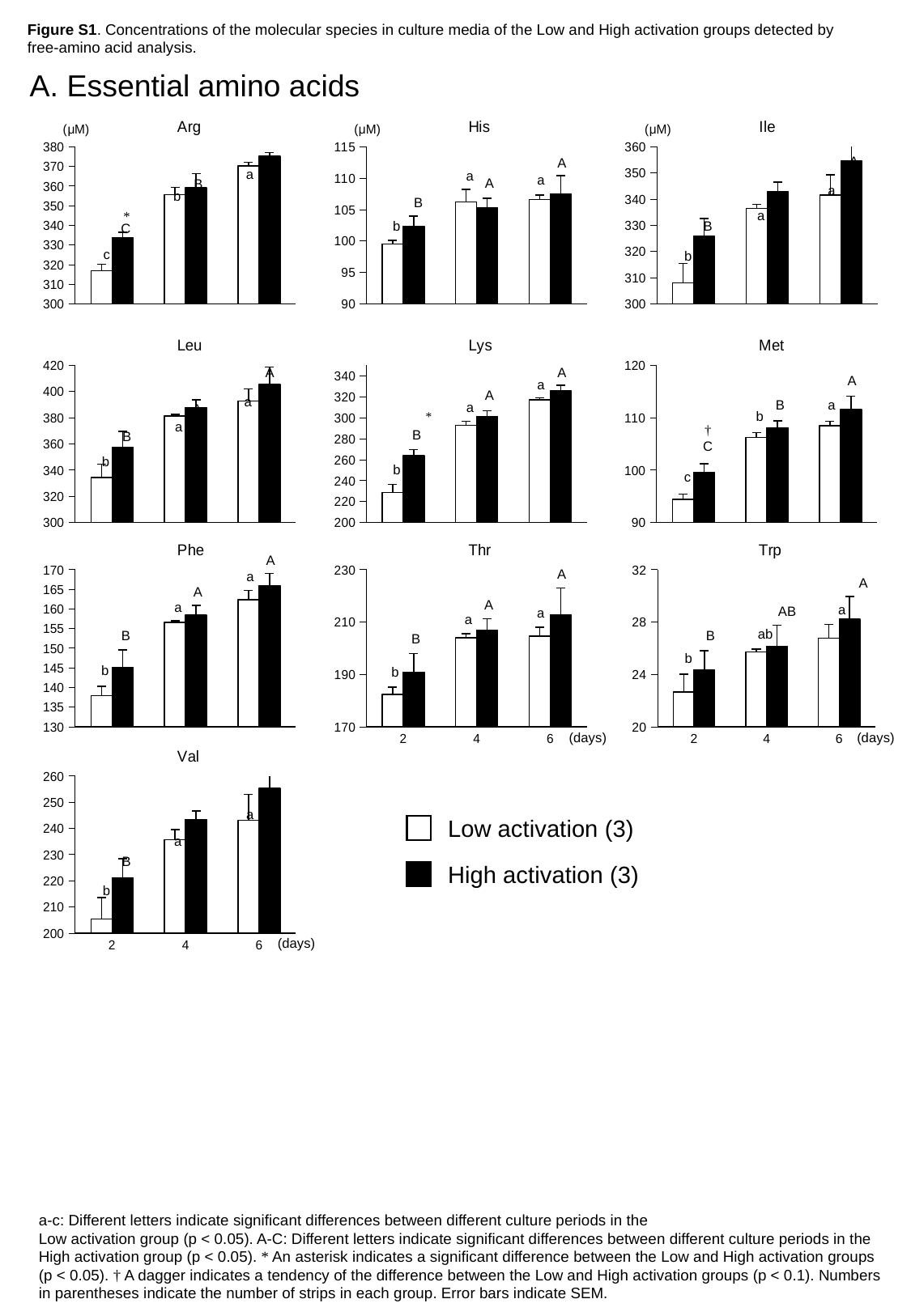

Figure S1. Concentrations of the molecular species in culture media of the Low and High activation groups detected by free-amino acid analysis.
A. Essential amino acids
### Chart: Arg
| Category | Few (3) | Many (3) |
|---|---|---|
| 2 | 316.84125 | 333.88175 |
| 4 | 355.66349999999994 | 359.08225 |
| 6 | 370.26574999999997 | 375.22049999999996 |A
a
B
b
*
C
c
### Chart: His
| Category | Few (3) | Many (3) |
|---|---|---|
| 2 | 99.56875000000001 | 102.354 |
| 4 | 106.21775000000001 | 105.242 |
| 6 | 106.658 | 107.519 |A
a
a
A
B
b
### Chart: Ile
| Category | Few (3) | Many (3) |
|---|---|---|
| 2 | 308.0205 | 325.89875 |
| 4 | 336.5545 | 342.77025 |
| 6 | 341.57899999999995 | 354.67125000000004 |A
a
A
a
B
b
### Chart: Leu
| Category | Few (3) | Many (3) |
|---|---|---|
| 2 | 334.289 | 357.44800000000004 |
| 4 | 381.44325000000003 | 387.797 |
| 6 | 392.96125 | 405.41125000000005 |A
a
A
a
B
b
### Chart: Lys
| Category | Few (3) | Many (3) |
|---|---|---|
| 2 | 228.94150000000002 | 263.74399999999997 |
| 4 | 293.00250000000005 | 301.34075 |
| 6 | 317.02324999999996 | 325.53925000000004 |A
a
A
a
B
b
### Chart: Met
| Category | Few (3) | Many (3) |
|---|---|---|
| 2 | 94.45974999999999 | 99.599 |
| 4 | 106.23275000000001 | 108.0895 |
| 6 | 108.51025 | 111.58625 |A
B
a
b
†
C
c
### Chart: Phe
| Category | Few (3) | Many (3) |
|---|---|---|
| 2 | 137.879 | 145.09050000000002 |
| 4 | 156.53799999999998 | 158.47375000000002 |
| 6 | 162.31825 | 165.909 |A
a
A
a
B
b
### Chart: Thr
| Category | Few (3) | Many (3) |
|---|---|---|
| 2 | 182.3935 | 190.88774999999998 |
| 4 | 204.02025 | 206.88449999999997 |
| 6 | 204.59775 | 212.73175 |A
A
a
a
B
b
### Chart: Trp
| Category | Few (3) | Many (3) |
|---|---|---|
| 2 | 22.652 | 24.3545 |
| 4 | 25.72075 | 26.144000000000002 |
| 6 | 26.77725 | 28.228250000000003 |A
a
AB
ab
B
b
(days)
(days)
### Chart: Val
| Category | Few (3) | Many (3) |
|---|---|---|
| 2 | 205.52775000000005 | 221.155 |
| 4 | 235.71000000000004 | 243.4825 |
| 6 | 243.06074999999998 | 255.29625 |A
a
A
a
B
b
Low activation (3)
High activation (3)
(days)
a-c: Different letters indicate significant differences between different culture periods in the
Low activation group (p < 0.05). A-C: Different letters indicate significant differences between different culture periods in the High activation group (p < 0.05). * An asterisk indicates a significant difference between the Low and High activation groups (p < 0.05). † A dagger indicates a tendency of the difference between the Low and High activation groups (p < 0.1). Numbers in parentheses indicate the number of strips in each group. Error bars indicate SEM.

## Slide 2
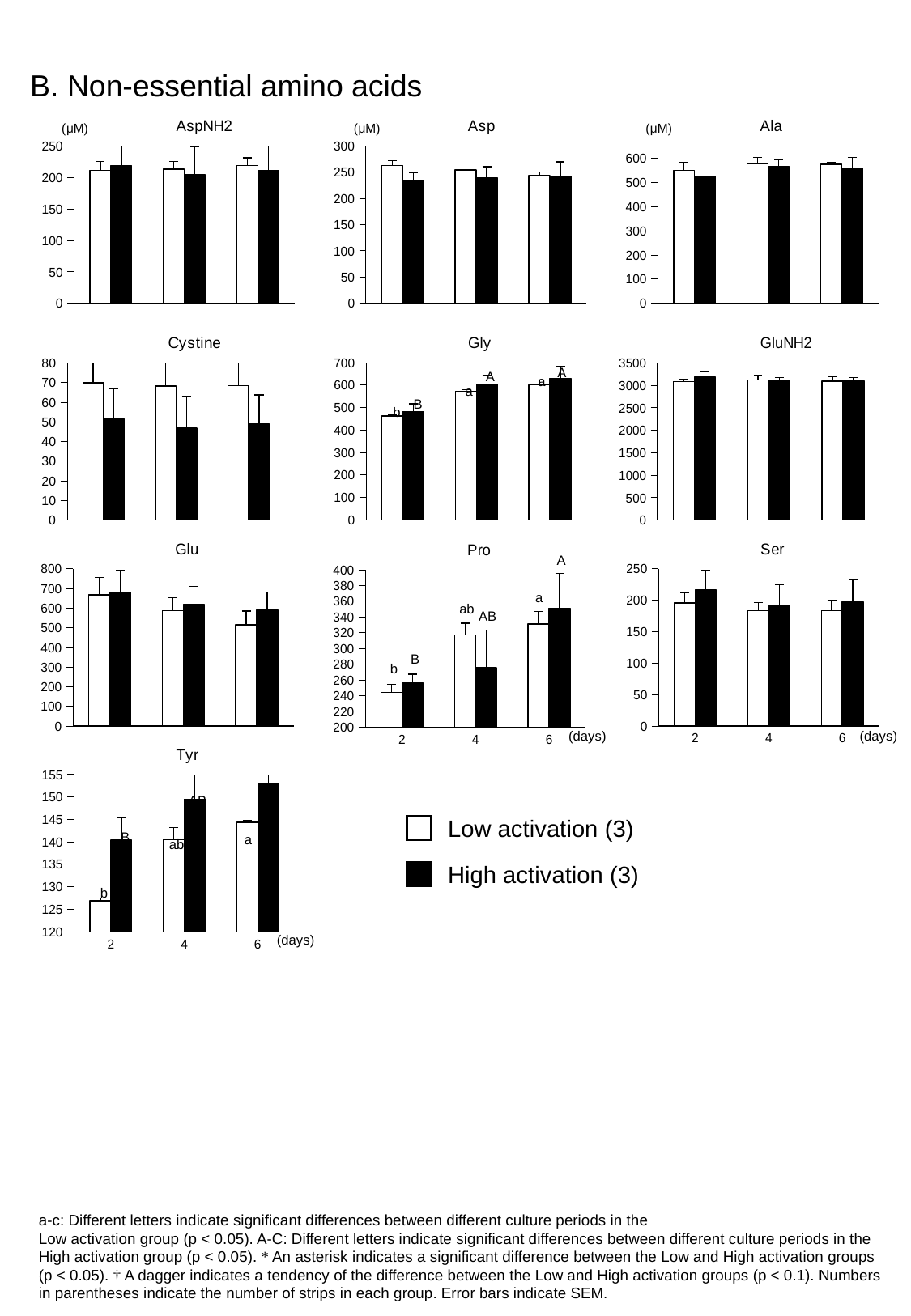

B. Non-essential amino acids
### Chart: AspNH2
| Category | Few (3) | Many (3) |
|---|---|---|
| 2 | 211.641 | 219.0385 |
| 4 | 213.367 | 204.27625 |
| 6 | 219.182 | 210.7275 |
### Chart: Ala
| Category | Few (3) | Many (3) |
|---|---|---|
| 2 | 549.19875 | 525.7197500000001 |
| 4 | 577.88475 | 566.3147499999999 |
| 6 | 574.795 | 560.60325 |
### Chart: Asp
| Category | Few (3) | Many (3) |
|---|---|---|
| 2 | 262.74525 | 233.16075 |
| 4 | 254.6455 | 239.67125000000001 |
| 6 | 243.424 | 241.76399999999998 |
### Chart: Gly
| Category | Few (3) | Many (3) |
|---|---|---|
| 2 | 462.30749999999995 | 483.24950000000007 |
| 4 | 572.89375 | 604.66475 |
| 6 | 602.191 | 630.89825 |A
A
a
a
B
b
### Chart: Cystine
| Category | Few (3) | Many (3) |
|---|---|---|
| 2 | 69.89450000000001 | 51.519000000000005 |
| 4 | 68.2695 | 46.77824999999999 |
| 6 | 68.4445 | 49.028 |
### Chart: GluNH2
| Category | Few (3) | Many (3) |
|---|---|---|
| 2 | 3085.8275 | 3183.2549999999997 |
| 4 | 3119.595 | 3112.3999999999996 |
| 6 | 3092.72 | 3095.295 |
### Chart: Glu
| Category | Few (3) | Many (3) |
|---|---|---|
| 2 | 668.1727500000001 | 682.9722499999999 |
| 4 | 586.91925 | 618.9195 |
| 6 | 515.347 | 591.61575 |
### Chart: Ser
| Category | Few (3) | Many (3) |
|---|---|---|
| 2 | 195.962 | 216.63125 |
| 4 | 183.73925 | 191.24125 |
| 6 | 183.58975 | 197.68549999999996 |
### Chart: Pro
| Category | Few (3) | Many (3) |
|---|---|---|
| 2 | 243.871 | 256.49525 |
| 4 | 317.2185 | 275.545 |
| 6 | 331.14925 | 351.15825 |A
a
ab
AB
B
b
(days)
(days)
### Chart: Tyr
| Category | Few (3) | Many (3) |
|---|---|---|
| 2 | 126.85375 | 140.403 |
| 4 | 140.51624999999999 | 149.5465 |
| 6 | 144.367 | 152.97525 |A
AB
B
a
ab
b
Low activation (3)
High activation (3)
(days)
a-c: Different letters indicate significant differences between different culture periods in the
Low activation group (p < 0.05). A-C: Different letters indicate significant differences between different culture periods in the High activation group (p < 0.05). * An asterisk indicates a significant difference between the Low and High activation groups (p < 0.05). † A dagger indicates a tendency of the difference between the Low and High activation groups (p < 0.1). Numbers in parentheses indicate the number of strips in each group. Error bars indicate SEM.

## Slide 3
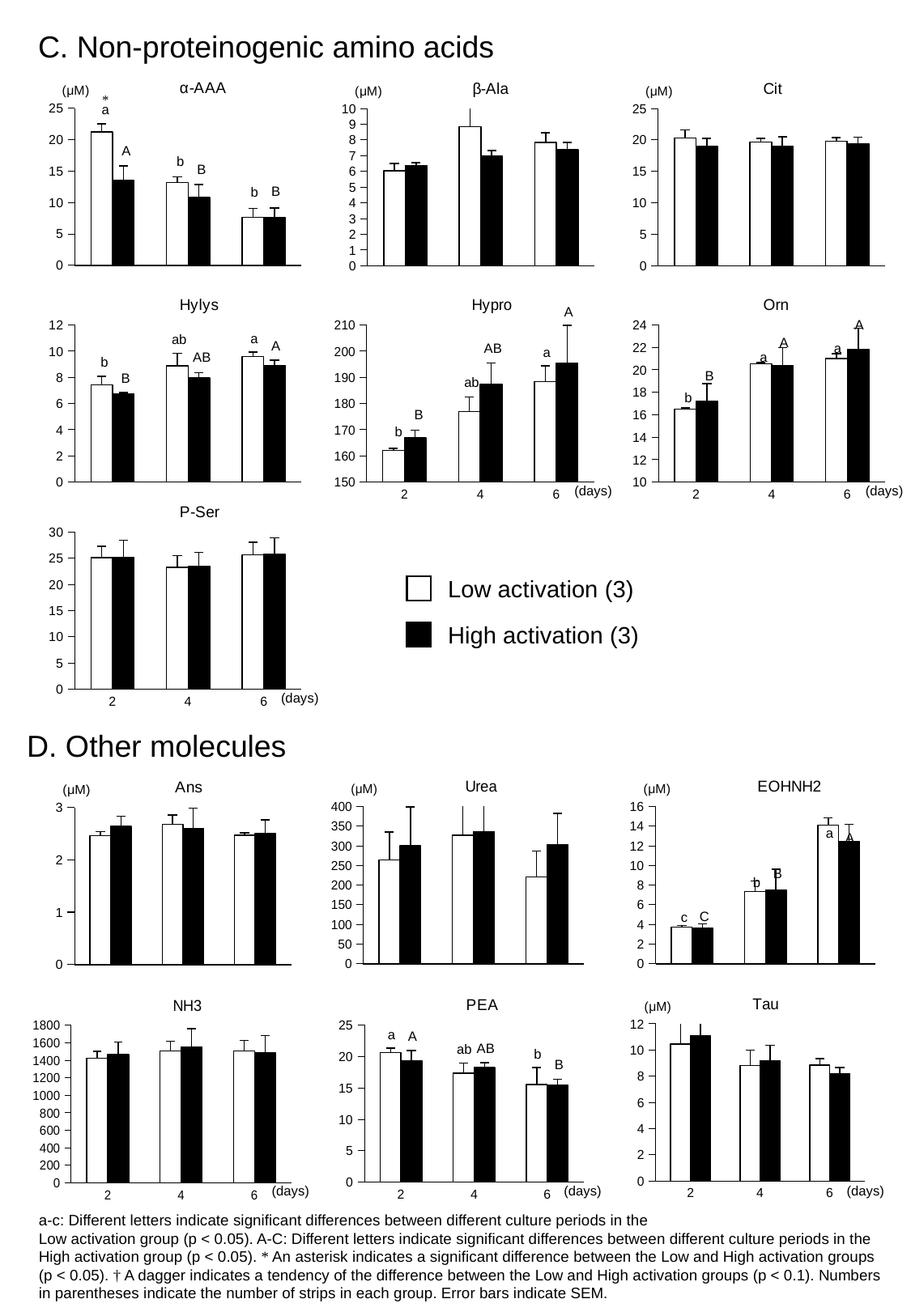

C. Non-proteinogenic amino acids
### Chart: α-AAA
| Category | Few (3) | Many (3) |
|---|---|---|
| 2 | 21.2375 | 13.46275 |
| 4 | 13.207749999999999 | 10.786000000000001 |
| 6 | 7.656749999999999 | 7.601749999999999 |*
a
A
b
B
B
b
### Chart: β-Ala
| Category | Few (3) | Many (3) |
|---|---|---|
| 2 | 6.044 | 6.365750000000001 |
| 4 | 8.844499999999998 | 6.976249999999999 |
| 6 | 7.837750000000001 | 7.374 |
### Chart: Cit
| Category | Few (3) | Many (3) |
|---|---|---|
| 2 | 20.318749999999998 | 19.040999999999997 |
| 4 | 19.6705 | 19.02925 |
| 6 | 19.815749999999998 | 19.377750000000002 |
### Chart: Hylys
| Category | Few (3) | Many (3) |
|---|---|---|
| 2 | 7.434249999999999 | 6.742250000000001 |
| 4 | 8.87075 | 7.9552499999999995 |
| 6 | 9.58425 | 8.90975 |a
ab
A
AB
b
B
### Chart: Hypro
| Category | Few (3) | Many (3) |
|---|---|---|
| 2 | 162.1155 | 166.90775 |
| 4 | 176.93025 | 187.39225 |
| 6 | 188.30899999999997 | 195.41125 |A
AB
a
ab
B
b
### Chart: Orn
| Category | Few (3) | Many (3) |
|---|---|---|
| 2 | 16.50325 | 17.21525 |
| 4 | 20.521 | 20.3755 |
| 6 | 20.995 | 21.8275 |A
A
a
a
B
b
(days)
(days)
### Chart: P-Ser
| Category | Few (3) | Many (3) |
|---|---|---|
| 2 | 25.078 | 25.1645 |
| 4 | 23.289749999999998 | 23.525000000000002 |
| 6 | 25.681750000000005 | 25.763499999999997 |Low activation (3)
High activation (3)
(days)
D. Other molecules
### Chart: EOHNH2
| Category | Few (3) | Many (3) |
|---|---|---|
| 2 | 3.7244999999999995 | 3.6212500000000003 |
| 4 | 7.3465 | 7.4985 |
| 6 | 14.125749999999998 | 12.441000000000003 |a
A
B
b
C
c
### Chart: Urea
| Category | Few (3) | Many (3) |
|---|---|---|
| 2 | 264.6705 | 300.64475 |
| 4 | 327.2485 | 336.32275 |
| 6 | 220.43975 | 303.7955 |
### Chart: Ans
| Category | Few (3) | Many (3) |
|---|---|---|
| 2 | 2.4635 | 2.63925 |
| 4 | 2.6750000000000003 | 2.5995 |
| 6 | 2.465 | 2.501 |
### Chart: Tau
| Category | Few (3) | Many (3) |
|---|---|---|
| 2 | 10.46025 | 11.10375 |
| 4 | 8.82425 | 9.198749999999999 |
| 6 | 8.8585 | 8.189250000000001 |
### Chart: PEA
| Category | Few (3) | Many (3) |
|---|---|---|
| 2 | 20.58975 | 19.3265 |
| 4 | 17.344 | 18.250249999999998 |
| 6 | 15.512750000000002 | 15.46575 |a
A
AB
ab
b
B
### Chart: NH3
| Category | Few (3) | Many (3) |
|---|---|---|
| 2 | 1426.5875000000003 | 1465.9875 |
| 4 | 1505.2849999999999 | 1556.2124999999996 |
| 6 | 1510.2600000000002 | 1492.07 |(days)
(days)
(days)
a-c: Different letters indicate significant differences between different culture periods in the
Low activation group (p < 0.05). A-C: Different letters indicate significant differences between different culture periods in the High activation group (p < 0.05). * An asterisk indicates a significant difference between the Low and High activation groups (p < 0.05). † A dagger indicates a tendency of the difference between the Low and High activation groups (p < 0.1). Numbers in parentheses indicate the number of strips in each group. Error bars indicate SEM.

## Slide 4
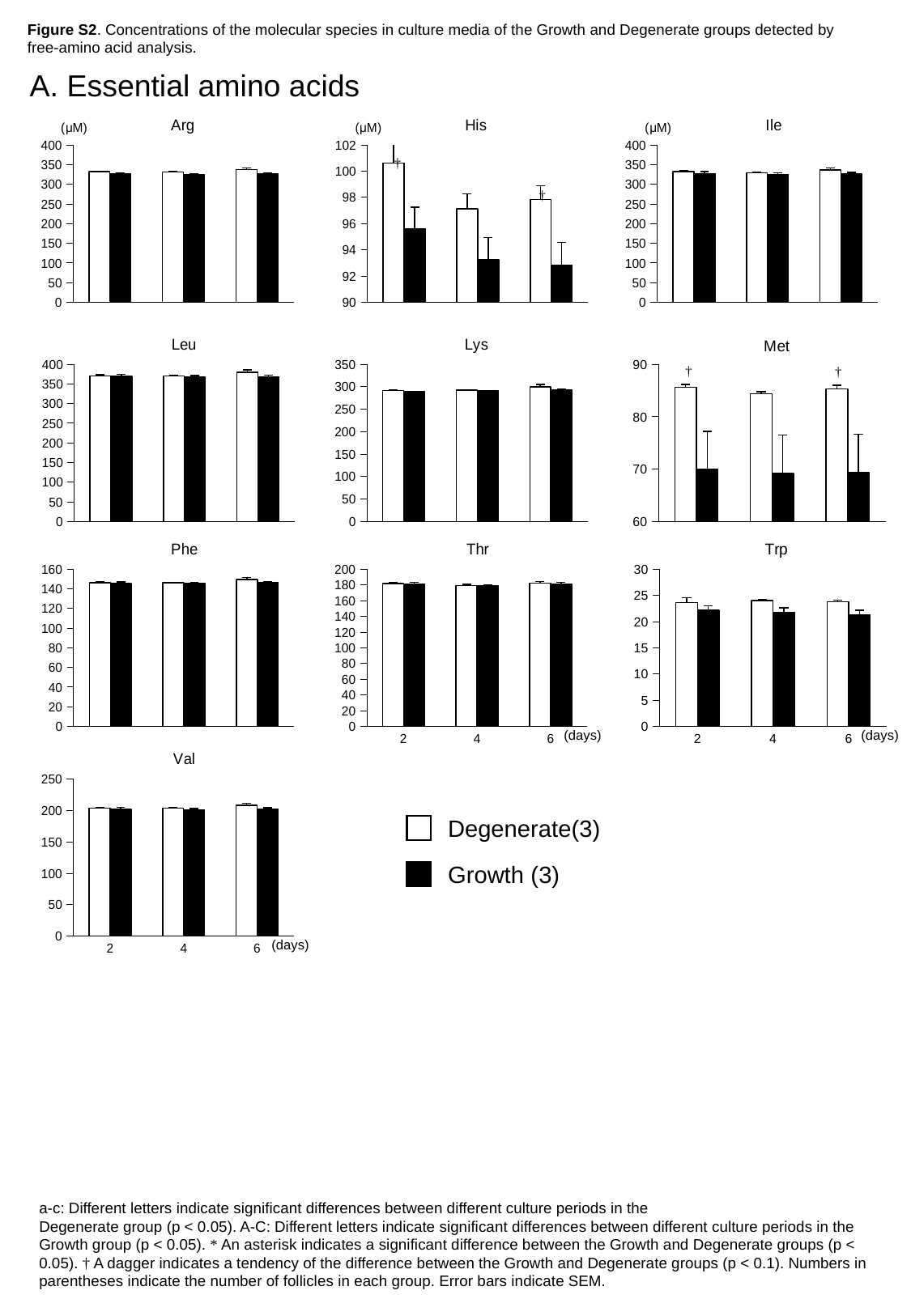

Figure S2. Concentrations of the molecular species in culture media of the Growth and Degenerate groups detected by free-amino acid analysis.
A. Essential amino acids
### Chart: Arg
| Category | Small (3) | Large (3) |
|---|---|---|
| 2 | 332.4725 | 326.4515 |
| 4 | 331.0845 | 325.23275000000007 |
| 6 | 336.9855 | 326.3445 |
### Chart: His
| Category | Small (3) | Large (3) |
|---|---|---|
| 2 | 100.60575 | 95.616 |
| 4 | 97.13999999999999 | 93.22825 |
| 6 | 97.8395 | 92.82175 |†
†
### Chart: Ile
| Category | Small (3) | Large (3) |
|---|---|---|
| 2 | 332.06525 | 327.70099999999996 |
| 4 | 329.487 | 325.2915 |
| 6 | 336.9005 | 326.016 |
### Chart: Leu
| Category | Small (3) | Large (3) |
|---|---|---|
| 2 | 370.3010000000001 | 368.97800000000007 |
| 4 | 370.49399999999997 | 366.98025 |
| 6 | 379.84175000000005 | 368.46975000000003 |
### Chart: Lys
| Category | Small (3) | Large (3) |
|---|---|---|
| 2 | 291.38425 | 290.00649999999996 |
| 4 | 292.79675000000003 | 290.87899999999996 |
| 6 | 300.04425 | 293.1765 |
### Chart: Met
| Category | Small (3) | Large (3) |
|---|---|---|
| 2 | 85.61225000000002 | 69.95825 |
| 4 | 84.348 | 69.19 |
| 6 | 85.28275000000001 | 69.41825 |†
†
### Chart: Phe
| Category | Small (3) | Large (3) |
|---|---|---|
| 2 | 146.34699999999998 | 145.2285 |
| 4 | 146.19375 | 145.573 |
| 6 | 149.6575 | 146.78324999999998 |
### Chart: Thr
| Category | Small (3) | Large (3) |
|---|---|---|
| 2 | 181.84324999999998 | 181.207 |
| 4 | 179.18574999999998 | 178.92525 |
| 6 | 182.4565 | 181.00825 |
### Chart: Trp
| Category | Small (3) | Large (3) |
|---|---|---|
| 2 | 23.604 | 22.194000000000003 |
| 4 | 24.017 | 21.80275 |
| 6 | 23.815749999999998 | 21.302249999999997 |(days)
(days)
### Chart: Val
| Category | Small (3) | Large (3) |
|---|---|---|
| 2 | 203.60924999999997 | 201.56275000000002 |
| 4 | 203.70950000000002 | 200.98850000000002 |
| 6 | 208.07725000000002 | 202.59675000000001 |Degenerate(3)
Growth (3)
(days)
a-c: Different letters indicate significant differences between different culture periods in the
Degenerate group (p < 0.05). A-C: Different letters indicate significant differences between different culture periods in the Growth group (p < 0.05). * An asterisk indicates a significant difference between the Growth and Degenerate groups (p < 0.05). † A dagger indicates a tendency of the difference between the Growth and Degenerate groups (p < 0.1). Numbers in parentheses indicate the number of follicles in each group. Error bars indicate SEM.

## Slide 5
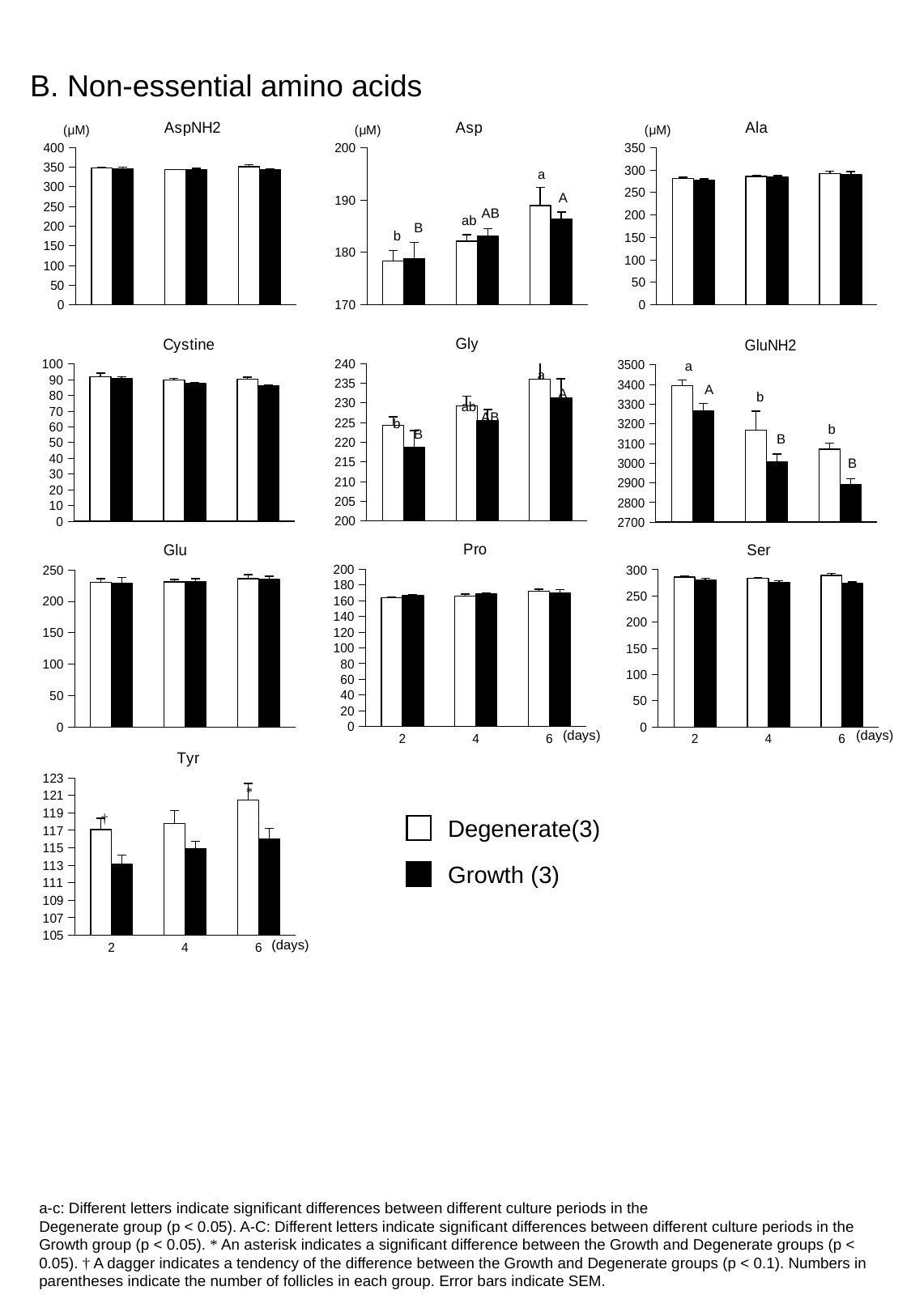

B. Non-essential amino acids
### Chart: AspNH2
| Category | Small (3) | Large (3) |
|---|---|---|
| 2 | 347.80775 | 346.46325 |
| 4 | 344.2435 | 344.578 |
| 6 | 351.468 | 344.44924999999995 |
### Chart: Asp
| Category | Small (3) | Large (3) |
|---|---|---|
| 2 | 178.3545 | 178.84725000000003 |
| 4 | 182.12275 | 183.10799999999998 |
| 6 | 188.94475000000003 | 186.35174999999995 |a
A
AB
ab
B
b
### Chart: Ala
| Category | Small (3) | Large (3) |
|---|---|---|
| 2 | 281.80899999999997 | 277.4 |
| 4 | 285.66249999999997 | 284.2 |
| 6 | 291.93125000000003 | 289.54925 |
### Chart: Gly
| Category | Small (3) | Large (3) |
|---|---|---|
| 2 | 224.38275000000002 | 218.62750000000003 |
| 4 | 229.2055 | 225.578 |
| 6 | 236.12749999999997 | 231.299 |a
A
ab
AB
b
B
### Chart: Cystine
| Category | Small (3) | Large (3) |
|---|---|---|
| 2 | 91.95125 | 90.87349999999999 |
| 4 | 89.72575000000002 | 87.49425000000001 |
| 6 | 90.42374999999998 | 85.98899999999999 |
### Chart: GluNH2
| Category | Small (3) | Large (3) |
|---|---|---|
| 2 | 3394.2099999999996 | 3265.5425 |
| 4 | 3168.8524999999995 | 3005.6725 |
| 6 | 3071.3525000000004 | 2890.8725 |a
A
b
b
B
B
### Chart: Pro
| Category | Small (3) | Large (3) |
|---|---|---|
| 2 | 163.47799999999998 | 166.43275 |
| 4 | 166.03549999999998 | 168.462 |
| 6 | 171.87325 | 169.62750000000003 |
### Chart: Ser
| Category | Small (3) | Large (3) |
|---|---|---|
| 2 | 285.9565 | 280.6755 |
| 4 | 284.15825 | 276.01425 |
| 6 | 289.0275 | 274.38275 |
### Chart: Glu
| Category | Small (3) | Large (3) |
|---|---|---|
| 2 | 230.3665 | 228.87875 |
| 4 | 230.98325 | 231.347 |
| 6 | 236.2275 | 234.571 |(days)
(days)
### Chart: Tyr
| Category | Small (3) | Large (3) |
|---|---|---|
| 2 | 117.068 | 113.1185 |
| 4 | 117.76774999999999 | 114.895 |
| 6 | 120.4785 | 116.0465 |*
†
Degenerate(3)
Growth (3)
(days)
a-c: Different letters indicate significant differences between different culture periods in the
Degenerate group (p < 0.05). A-C: Different letters indicate significant differences between different culture periods in the Growth group (p < 0.05). * An asterisk indicates a significant difference between the Growth and Degenerate groups (p < 0.05). † A dagger indicates a tendency of the difference between the Growth and Degenerate groups (p < 0.1). Numbers in parentheses indicate the number of follicles in each group. Error bars indicate SEM.

## Slide 6
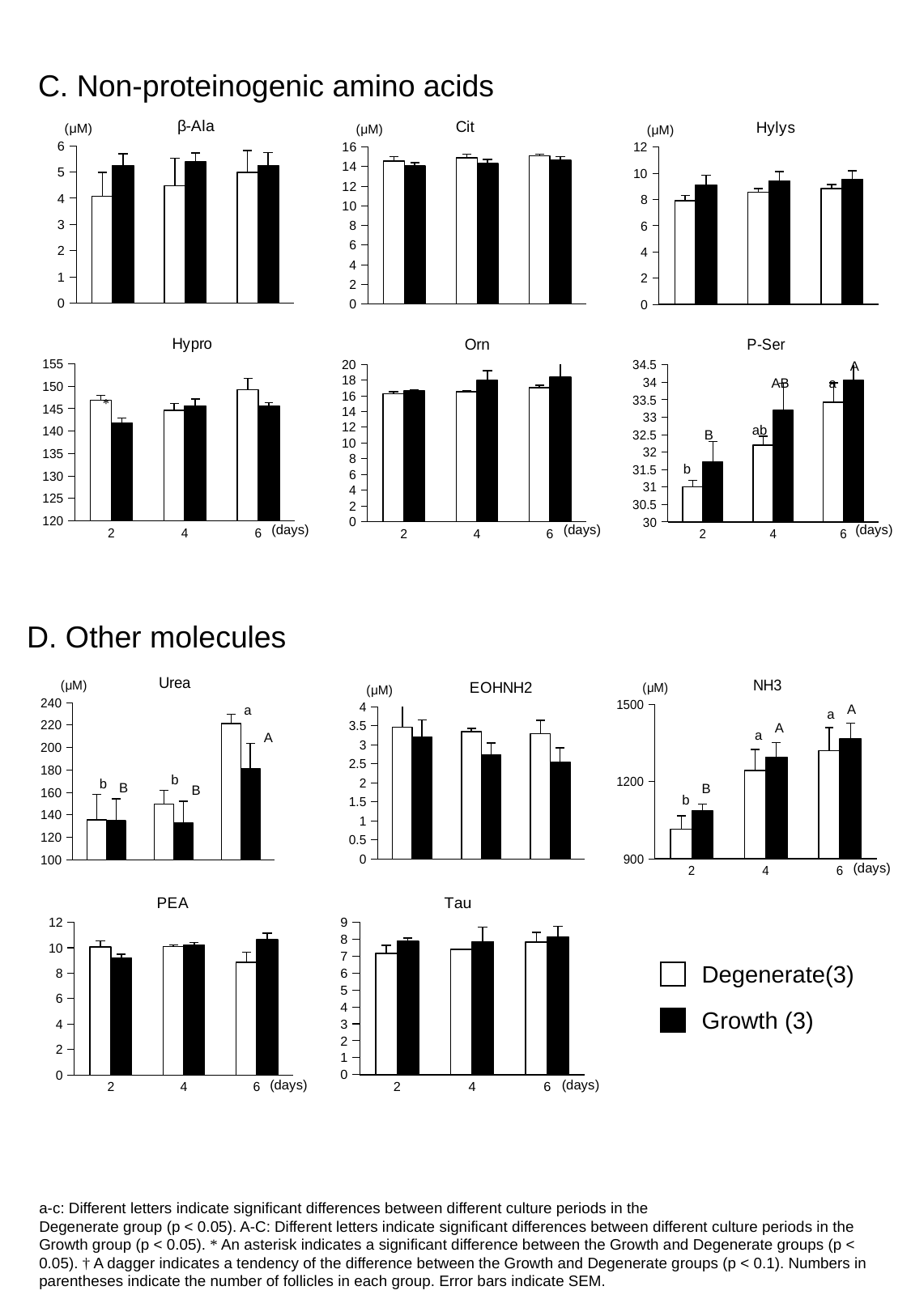

C. Non-proteinogenic amino acids
### Chart: β-Ala
| Category | Small (3) | Large (3) |
|---|---|---|
| 2 | 4.077750000000001 | 5.247000000000001 |
| 4 | 4.47325 | 5.39225 |
| 6 | 4.99325 | 5.25525 |
### Chart: Cit
| Category | Small (3) | Large (3) |
|---|---|---|
| 2 | 14.57175 | 14.065750000000001 |
| 4 | 14.884500000000001 | 14.306 |
| 6 | 15.077 | 14.628 |
### Chart: Hylys
| Category | Small (3) | Large (3) |
|---|---|---|
| 2 | 7.92125 | 9.12775 |
| 4 | 8.546000000000001 | 9.430000000000001 |
| 6 | 8.83625 | 9.5565 |
### Chart: Hypro
| Category | Small (3) | Large (3) |
|---|---|---|
| 2 | 146.89200000000002 | 141.81925 |
| 4 | 144.6395 | 145.58075 |
| 6 | 149.25725 | 145.563 |*
### Chart: Orn
| Category | Small (3) | Large (3) |
|---|---|---|
| 2 | 16.28775 | 16.61175 |
| 4 | 16.520500000000002 | 17.95975 |
| 6 | 17.0415 | 18.3605 |
### Chart: P-Ser
| Category | Small (3) | Large (3) |
|---|---|---|
| 2 | 31.013 | 31.719000000000005 |
| 4 | 32.198750000000004 | 33.2005 |
| 6 | 33.43025 | 34.053000000000004 |A
a
AB
ab
B
b
(days)
(days)
(days)
D. Other molecules
### Chart: Urea
| Category | Small (3) | Large (3) |
|---|---|---|
| 2 | 135.59025 | 134.89124999999999 |
| 4 | 149.7515 | 133.14000000000001 |
| 6 | 221.27875000000003 | 181.28674999999998 |a
A
b
b
B
B
### Chart: NH3
| Category | Small (3) | Large (3) |
|---|---|---|
| 2 | 1014.7240000000002 | 1086.36275 |
| 4 | 1242.67825 | 1295.9245 |
| 6 | 1319.9470000000001 | 1365.4359999999997 |A
a
A
a
B
b
### Chart: EOHNH2
| Category | Small (3) | Large (3) |
|---|---|---|
| 2 | 3.46425 | 3.2102500000000003 |
| 4 | 3.34275 | 2.7345 |
| 6 | 3.29325 | 2.5517500000000006 |(days)
### Chart: Tau
| Category | Small (3) | Large (3) |
|---|---|---|
| 2 | 7.156999999999999 | 7.8790000000000004 |
| 4 | 7.3875 | 7.85475 |
| 6 | 7.81425 | 8.13 |
### Chart: PEA
| Category | Small (3) | Large (3) |
|---|---|---|
| 2 | 10.07125 | 9.20025 |
| 4 | 10.1105 | 10.2135 |
| 6 | 8.8535 | 10.637 |Degenerate(3)
Growth (3)
(days)
(days)
a-c: Different letters indicate significant differences between different culture periods in the
Degenerate group (p < 0.05). A-C: Different letters indicate significant differences between different culture periods in the Growth group (p < 0.05). * An asterisk indicates a significant difference between the Growth and Degenerate groups (p < 0.05). † A dagger indicates a tendency of the difference between the Growth and Degenerate groups (p < 0.1). Numbers in parentheses indicate the number of follicles in each group. Error bars indicate SEM.

## Slide 7
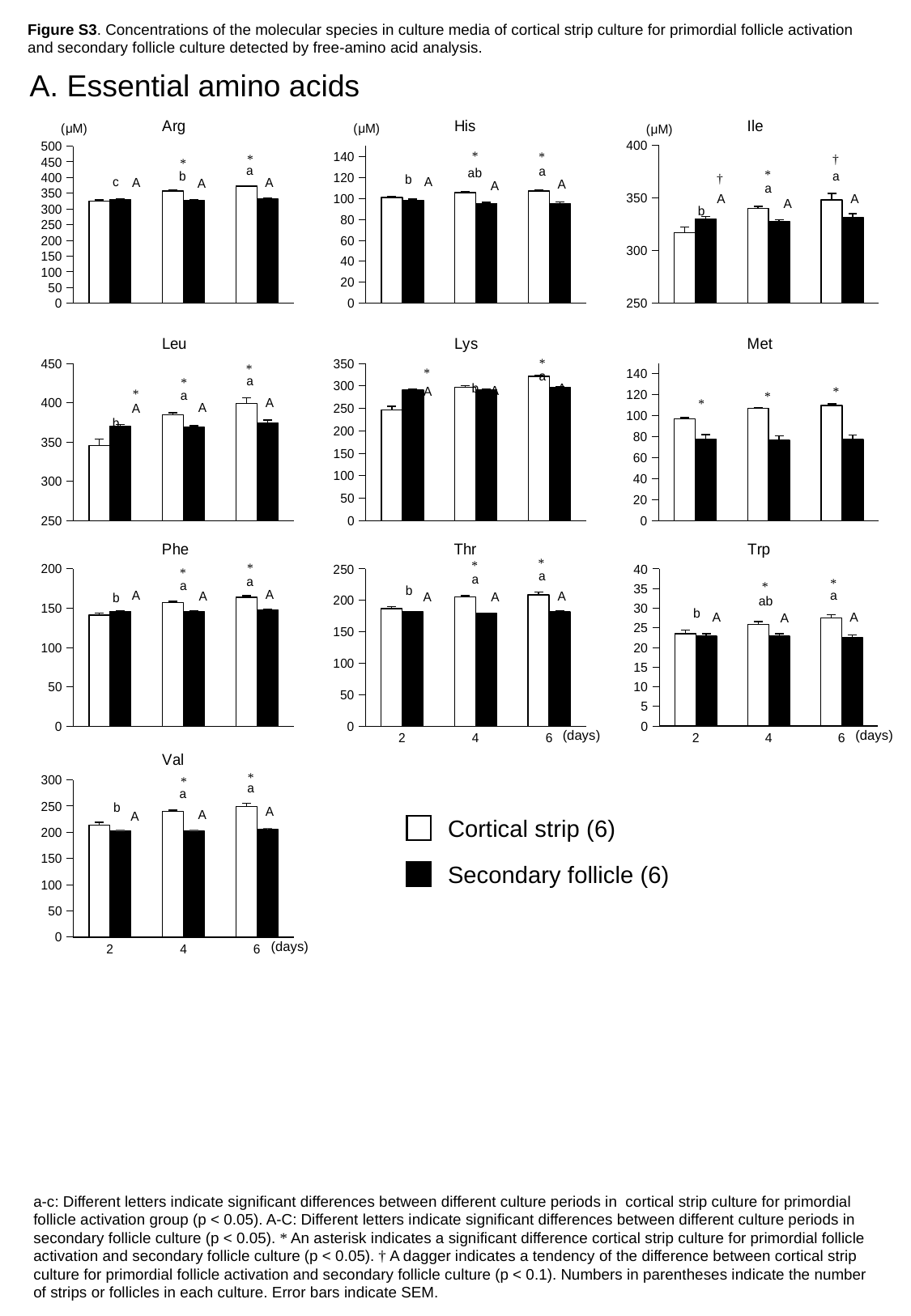

Figure S3. Concentrations of the molecular species in culture media of cortical strip culture for primordial follicle activation and secondary follicle culture detected by free-amino acid analysis.
A. Essential amino acids
### Chart: Arg
| Category | Primordial (6) | Secondary (6) |
|---|---|---|
| 2 | 325.36150000000004 | 329.46200000000005 |
| 4 | 357.372875 | 328.15862500000003 |
| 6 | 372.74312499999996 | 331.665 |
### Chart: His
| Category | Primordial (6) | Secondary (6) |
|---|---|---|
| 2 | 100.96137500000002 | 98.11087500000001 |
| 4 | 105.729875 | 95.184125 |
| 6 | 107.0885 | 95.33062500000001 |
### Chart: Ile
| Category | Primordial (6) | Secondary (6) |
|---|---|---|
| 2 | 316.959625 | 329.883125 |
| 4 | 339.66237500000005 | 327.38925 |
| 6 | 348.125125 | 331.45825 |
### Chart: Met
| Category | Primordial (6) | Secondary (6) |
|---|---|---|
| 2 | 97.029375 | 77.78525 |
| 4 | 107.16112500000001 | 76.76899999999999 |
| 6 | 110.04825000000001 | 77.3505 |
### Chart: Leu
| Category | Primordial (6) | Secondary (6) |
|---|---|---|
| 2 | 345.8685 | 369.63950000000006 |
| 4 | 384.6201250000001 | 368.73712499999993 |
| 6 | 399.18625000000003 | 374.15575000000007 |
### Chart: Lys
| Category | Primordial (6) | Secondary (6) |
|---|---|---|
| 2 | 246.34275000000002 | 290.69537499999996 |
| 4 | 297.171625 | 291.837875 |
| 6 | 321.28125 | 296.610375 |
### Chart: Phe
| Category | Primordial (6) | Secondary (6) |
|---|---|---|
| 2 | 141.48475 | 145.78775 |
| 4 | 157.505875 | 145.883375 |
| 6 | 164.11362499999998 | 148.220375 |
### Chart: Thr
| Category | Primordial (6) | Secondary (6) |
|---|---|---|
| 2 | 186.640625 | 181.52512499999997 |
| 4 | 205.452375 | 179.05550000000002 |
| 6 | 208.66475 | 181.73237500000002 |
### Chart: Trp
| Category | Primordial (6) | Secondary (6) |
|---|---|---|
| 2 | 23.503250000000005 | 22.899 |
| 4 | 25.932374999999997 | 22.909875 |
| 6 | 27.502750000000002 | 22.558999999999997 |(days)
(days)
### Chart: Val
| Category | Primordial (6) | Secondary (6) |
|---|---|---|
| 2 | 213.341375 | 202.586 |
| 4 | 239.59625000000003 | 202.34900000000002 |
| 6 | 249.17849999999999 | 205.33700000000002 |Cortical strip (6)
Secondary follicle (6)
(days)
a-c: Different letters indicate significant differences between different culture periods in cortical strip culture for primordial follicle activation group (p < 0.05). A-C: Different letters indicate significant differences between different culture periods in secondary follicle culture (p < 0.05). * An asterisk indicates a significant difference cortical strip culture for primordial follicle activation and secondary follicle culture (p < 0.05). † A dagger indicates a tendency of the difference between cortical strip culture for primordial follicle activation and secondary follicle culture (p < 0.1). Numbers in parentheses indicate the number of strips or follicles in each culture. Error bars indicate SEM.

## Slide 8
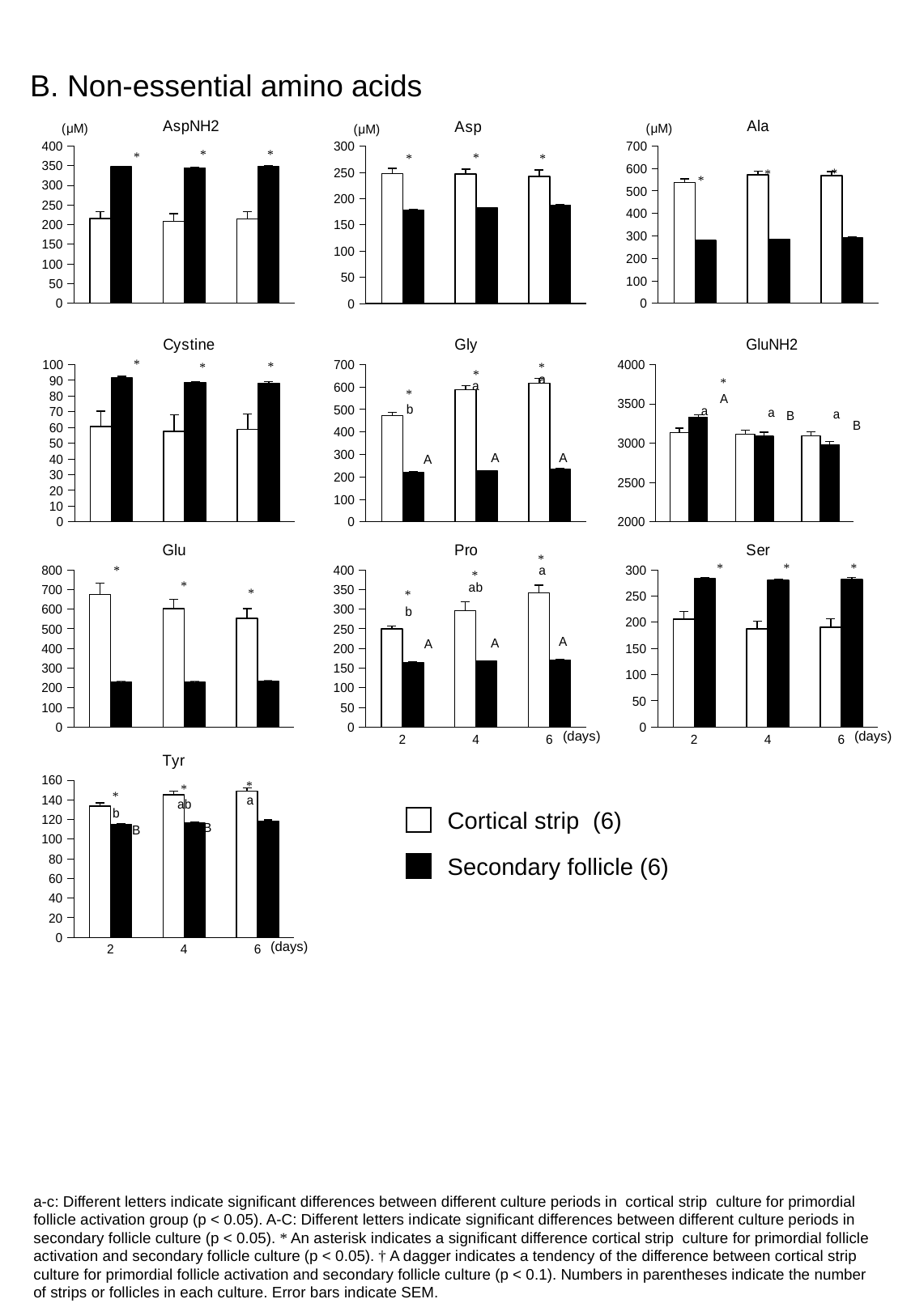

B. Non-essential amino acids
### Chart: AspNH2
| Category | Primordial (6) | Secondary (6) |
|---|---|---|
| 2 | 215.33975 | 347.13550000000004 |
| 4 | 208.82162499999995 | 344.41075 |
| 6 | 214.95475000000002 | 347.958625 |
### Chart: Ala
| Category | Primordial (6) | Secondary (6) |
|---|---|---|
| 2 | 537.45925 | 279.6045 |
| 4 | 572.09975 | 284.93125 |
| 6 | 567.699125 | 290.74025000000006 |
### Chart: Asp
| Category | Primordial (6) | Secondary (6) |
|---|---|---|
| 2 | 247.953 | 178.600875 |
| 4 | 247.15837500000006 | 182.61537499999997 |
| 6 | 242.59400000000002 | 187.64825 |
### Chart: GluNH2
| Category | Primordial (6) | Secondary (6) |
|---|---|---|
| 2 | 3134.54125 | 3329.87625 |
| 4 | 3115.9974999999995 | 3087.2625000000003 |
| 6 | 3094.0075 | 2981.1124999999997 |
### Chart: Cystine
| Category | Primordial (6) | Secondary (6) |
|---|---|---|
| 2 | 60.70675 | 91.412375 |
| 4 | 57.523875 | 88.61000000000001 |
| 6 | 58.736250000000005 | 88.20637499999998 |
### Chart: Gly
| Category | Primordial (6) | Secondary (6) |
|---|---|---|
| 2 | 472.7784999999999 | 221.505125 |
| 4 | 588.77925 | 227.39175 |
| 6 | 616.544625 | 233.71325 |
### Chart: Glu
| Category | Primordial (6) | Secondary (6) |
|---|---|---|
| 2 | 675.5725 | 229.62262499999997 |
| 4 | 602.9193750000001 | 231.165125 |
| 6 | 553.481375 | 235.39925000000002 |
### Chart: Ser
| Category | Primordial (6) | Secondary (6) |
|---|---|---|
| 2 | 206.29662499999998 | 283.316 |
| 4 | 187.49024999999997 | 280.08625 |
| 6 | 190.63762499999999 | 281.705125 |
### Chart: Pro
| Category | Primordial (6) | Secondary (6) |
|---|---|---|
| 2 | 250.18312500000002 | 164.955375 |
| 4 | 296.38175 | 167.24875 |
| 6 | 341.15374999999995 | 170.750375 |(days)
(days)
### Chart: Tyr
| Category | Primordial (6) | Secondary (6) |
|---|---|---|
| 2 | 133.62837499999998 | 115.09325000000001 |
| 4 | 145.031375 | 116.331375 |
| 6 | 148.671125 | 118.26249999999999 |Cortical strip (6)
Secondary follicle (6)
(days)
a-c: Different letters indicate significant differences between different culture periods in cortical strip culture for primordial follicle activation group (p < 0.05). A-C: Different letters indicate significant differences between different culture periods in secondary follicle culture (p < 0.05). * An asterisk indicates a significant difference cortical strip culture for primordial follicle activation and secondary follicle culture (p < 0.05). † A dagger indicates a tendency of the difference between cortical strip culture for primordial follicle activation and secondary follicle culture (p < 0.1). Numbers in parentheses indicate the number of strips or follicles in each culture. Error bars indicate SEM.

## Slide 9
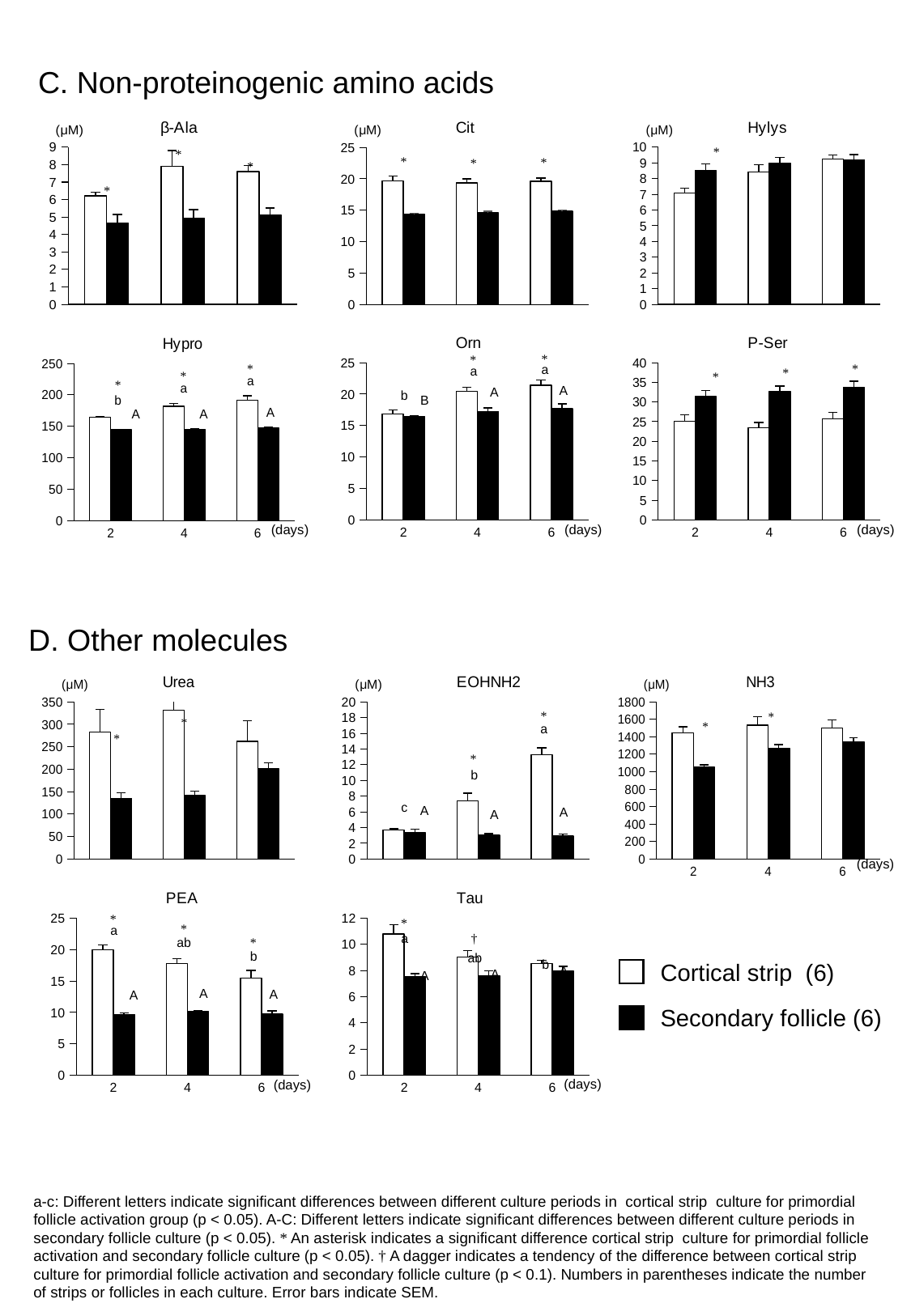

C. Non-proteinogenic amino acids
### Chart: β-Ala
| Category | Primordial (6) | Secondary (6) |
|---|---|---|
| 2 | 6.204875 | 4.662375 |
| 4 | 7.910374999999999 | 4.9327499999999995 |
| 6 | 7.605875 | 5.12425 |
### Chart: Hylys
| Category | Primordial (6) | Secondary (6) |
|---|---|---|
| 2 | 7.0882499999999995 | 8.5245 |
| 4 | 8.413 | 8.988000000000001 |
| 6 | 9.247 | 9.196375000000002 |
### Chart: Cit
| Category | Primordial (6) | Secondary (6) |
|---|---|---|
| 2 | 19.679875 | 14.318750000000001 |
| 4 | 19.349875 | 14.59525 |
| 6 | 19.59675 | 14.8525 |
### Chart: Orn
| Category | Primordial (6) | Secondary (6) |
|---|---|---|
| 2 | 16.85925 | 16.449749999999998 |
| 4 | 20.448249999999998 | 17.240125000000003 |
| 6 | 21.41125 | 17.700999999999997 |
### Chart: P-Ser
| Category | Primordial (6) | Secondary (6) |
|---|---|---|
| 2 | 25.12125 | 31.366 |
| 4 | 23.407375000000002 | 32.699625000000005 |
| 6 | 25.722624999999997 | 33.741625 |
### Chart: Hypro
| Category | Primordial (6) | Secondary (6) |
|---|---|---|
| 2 | 164.511625 | 144.355625 |
| 4 | 182.16125 | 145.110125 |
| 6 | 191.860125 | 147.410125 |(days)
(days)
(days)
D. Other molecules
### Chart: Urea
| Category | Primordial (6) | Secondary (6) |
|---|---|---|
| 2 | 282.657625 | 135.24075 |
| 4 | 331.7856249999999 | 141.44574999999998 |
| 6 | 262.117625 | 201.28275 |
### Chart: EOHNH2
| Category | Primordial (6) | Secondary (6) |
|---|---|---|
| 2 | 3.672875 | 3.3372499999999996 |
| 4 | 7.422499999999999 | 3.0386249999999997 |
| 6 | 13.283375 | 2.9225 |
### Chart: NH3
| Category | Primordial (6) | Secondary (6) |
|---|---|---|
| 2 | 1446.2875000000001 | 1050.543375 |
| 4 | 1530.74875 | 1269.301375 |
| 6 | 1501.165 | 1342.6915 |(days)
### Chart: Tau
| Category | Primordial (6) | Secondary (6) |
|---|---|---|
| 2 | 10.782000000000002 | 7.518 |
| 4 | 9.0115 | 7.621125 |
| 6 | 8.523875 | 7.972124999999999 |
### Chart: PEA
| Category | Primordial (6) | Secondary (6) |
|---|---|---|
| 2 | 19.958125 | 9.63575 |
| 4 | 17.797124999999998 | 10.161999999999999 |
| 6 | 15.489249999999998 | 9.74525 |Cortical strip (6)
Secondary follicle (6)
(days)
(days)
a-c: Different letters indicate significant differences between different culture periods in cortical strip culture for primordial follicle activation group (p < 0.05). A-C: Different letters indicate significant differences between different culture periods in secondary follicle culture (p < 0.05). * An asterisk indicates a significant difference cortical strip culture for primordial follicle activation and secondary follicle culture (p < 0.05). † A dagger indicates a tendency of the difference between cortical strip culture for primordial follicle activation and secondary follicle culture (p < 0.1). Numbers in parentheses indicate the number of strips or follicles in each culture. Error bars indicate SEM.
